# Preclinical Safety and Feasibility Study of Line-Field Confocal Optical Coherence Tomography for Ophthalmology Applications

**DOI:** 10.1167/tvst.15.7.1

**Published:** 2026-07-01

**Authors:** Emmanuel Crouzet, Jonas Ogien, Jean-Marc Dumollard, Chantal Perrache, Jean-Luc Perrot, Philippe Gain, Arnaud Dubois, Gilles Thuret

**Affiliations:** 1 Laboratoire de Biologie, ingénierie et imagerie pour l'Ophtalmologie, BiiO, Faculté de Santé, Campus Santé Innovations, Université de Saint-Etienne, Saint-Priest en Jarez, France; 2 DAMAE Medical, Paris, France; 3 Pathology Department, University Hospital of Saint-Etienne, Saint-Etienne Cedex, France; 4 Dermatology Department, University Hospital of Saint-Etienne, Saint-Etienne Cedex, France; 5 Ophthalmology Department, University Hospital of Saint-Etienne, Saint-Etienne Cedex, France; 6 Institut d'Optique Graduate School, IOGS, Palaiseau Cedex, France

**Keywords:** cornea, ophthalmology, confocal microscopy, optical coherence tomography (OCT), optical imaging

## Abstract

**Purpose:**

The purpose of this study was to demonstrate the first use of a commercially available, CE-marked line-field confocal optical coherence tomography (LC-OCT) in ophthalmology and assess its safety and feasibility for ocular imaging in rabbits, focusing on phototoxicity and tissue integrity.

**Methods:**

Five 14-week-old New Zealand White rabbits underwent corneal imaging using the DeepLive device (DAMAE Medical, Paris, France), originally developed for dermatologic applications. Imaging sessions lasted 5 to 10 minutes per eye, using illumination at a center wavelength of 820 nm and a power of 13.2 mW. Safety was evaluated in silico according to International Electrotechnical Commission (IEC) 60825-1 standards and in vivo through clinical assessments including slit-lamp examination, retinal imaging, and intraocular pressure (IOP) measurements at baseline, immediately after imaging, and on days 7 and 14. Histological analysis was performed day 14.

**Results:**

LC-OCT provided high-resolution three-dimensional imaging of the cornea (1.2 × 0.5 × 0.5 mm) and two-dimensional views of corneal layers, conjunctiva, and the nictitating membrane, revealing cellular details. In silico analysis confirmed that laser exposure remained below IEC safety limits. In vivo evaluations showed no corneal opacity, neovascularization, inflammation, or retinal alterations at any time point. Lens transparency remained normal (Lens Opacities Classification System [LOCS] III grade = 0), and IOP showed no treatment-related differences (*P* = 0.6713). Histology confirmed preservation of corneal and retinal structures.

**Conclusions:**

Ophthalmologic use of CE-marked LC-OCT demonstrated safe, effective imaging in rabbits without phototoxicity, supporting its potential for clinical ophthalmic applications.

**Translational Relevance:**

These findings provide preclinical safety evidence supporting LC-OCT as a noninvasive, high-resolution imaging modality for future clinical ophthalmology applications.

## Introduction

Advances in optical imaging technologies have transformed the field of ophthalmology by enabling high-resolution, noninvasive visualization of ocular structures.

Anterior segment optical coherence tomography (AS-OCT) is increasingly used in clinical practice for anterior segment imaging, providing high-resolution, cross-sectional visualization of the cornea, anterior chamber, and irido-corneal angle structures in a noninvasive manner.^1^ It is widely used in the diagnosis and management of corneal diseases, such as keratoconus^2^^,^^3^ and dystrophies,^4^ as well as for evaluating post-surgical outcomes in procedures like corneal transplantation and refractive surgery.^5^ Nevertheless, commercial AS-OCT devices do not resolve cellular-level details, limiting their utility for evaluating fine microstructural changes in corneal and conjunctival tissues.^6^

In vivo confocal microscopy (IVCM) has also long been a valuable tool, providing high-resolution imaging of the cornea and conjunctiva at the cellular level.^7^^,^^8^ It has been instrumental in diagnosing infectious keratitis,^9^^–^^11^ evaluating corneal dystrophies,^12^^,^^13^ and assessing nerve fiber alterations, in particular, in diabetic neuropathy.^8^^,^^14^ However, its limited imaging depth, small field of view, and inability to generate vertical cross-sectional 3D images constrain its clinical applications.^15^

Line field-confocal-OCT (LC-OCT) combines the advantages of both OCT and IVCM: the high-resolution capabilities of confocal microscopy with the depth imaging advantages and vertical sectioning capability of OCT.^16^^,^^17^ LC-OCT is a microscopic implementation of time-domain OCT with line illumination and detection.^18^ Interference images are continuously acquired by a line-scan camera, whereas the sample is scanned in depth for vertical imaging or laterally for horizontal imaging. The 3D images are obtained by combining depth scanning and lateral scanning. LC-OCT has already demonstrated significant utility in clinical dermatology, where it is used for noninvasive diagnosis and monitoring of skin disorders.^19^ Its ability to capture high-resolution, depth-resolved images of skin layers makes it a valuable tool for identifying basal cell carcinoma,^20^^,^^21^ squamous cell carcinoma and actinic keratosis,^22^^,^^23^ melanoma,^24^^–^^27^ cutaneous lymphoma,^28^ and inflammatory skin diseases.^29^^–^^31^ The real-time imaging capability of LC-OCT allows clinicians to assess skin microstructures and significantly reduce the need for biopsies.^32^ The success of LC-OCT in dermatology underscores its potential for broader applications in other medical fields, including ophthalmology.

The ability of LC-OCT to provide real-time, in vivo imaging of the anterior segment of the eye would open new possibilities for diagnostics, surgical guidance, and longitudinal monitoring of ocular diseases. However, its application in ophthalmology raises safety concerns, particularly regarding potential phototoxicity of the laser source used. LC-OCT operates at a wavelength of 820 nm — a range generally well-tolerated, even by photophobic patients.^33^ Nevertheless, safety standards for light-based imaging systems, such as those defined by the International Electrotechnical Commission (IEC 60825-1) for laser exposure, must be rigorously followed in order to prevent potential phototoxicity or thermal damage. To date, the specific impact of LC-OCT characteristics — including wavelength, power density, and exposure duration—on ocular tissues remains unknown, and no prior studies have investigated its safety or efficacy in ophthalmic applications.

In recent years, line-field OCT approaches, including both line-field and line-confocal implementations, have been explored for ophthalmic imaging, encompassing ex vivo studies and in vivo human applications.^34^^,^^35^

In this context, the present study investigates the in vivo application of a commercially available CE-marked LC-OCT system (DeepLive, manufactured by DAMAE Medical, Paris, France), initially developed for dermatological imaging, for ocular tissues. In addition, we conducted a focused safety assessment to eliminate any possibility of deleterious effects, particularly in anticipation of future human applications.

## Materials and Methods

### Line Field-Confocal-Optical Coherence Tomography

**Figure 1.**
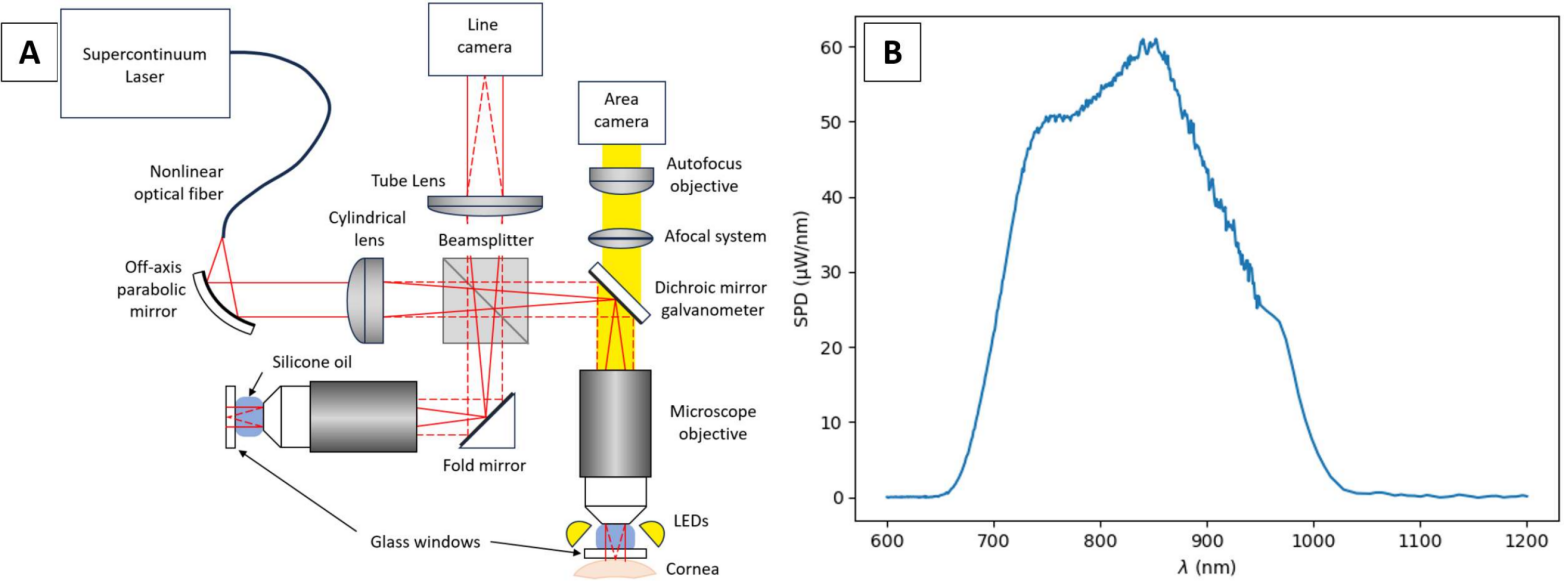
(**A**) Schematic diagram of the LC-OCT system. (**B**) Spectral power density of the supercontinuum laser illumination, at the level of the sample.

The LC-OCT system used for corneal imaging DeepLive. The LC-OCT optical setup included in the DeepLive device has been previously described in detail.^19^ In summary, it consisted of a Linnik interferometer, with illumination by a fibered supercontinuum laser, collimated and focused along a line on the sample by means of a cylindrical lens of focal length 50 mm and a microscope objective (20 ×, NA = 0.5, silicone-oil immersed) resulting in a line of 1.2 mm × 1.3 µm × 15 µm (lateral × vertical × depth) at the level of the sample. A fused silica glass window (diameter = 5.0 mm and thickness = 0.5 mm) with a flatness characterized by an angular deviation below 3′ (Fichou, Fresnes, France), was positioned under the objective and put in contact with the sample to be imaged to stabilize it and provide contact imaging without the need for adjusting the working distance of the objective. The light backscattered by the sample was recombined with the light coming from the reference arm of the interferometer, and an interference image was formed on a line-scan camera by means of a tube lens of focal length 150 mm. A schematic diagram of the LC-OCT system was provided Figure 1A.

The field of view provided by the DeepLive device was 1.2 mm × 0.4 mm (lateral × depth) in the vertical imaging mode, and 1.2 mm × 0.5 mm in the horizontal imaging mode. A 3D mode also allowed to stack horizontal images during depth scanning, resulting in 3D images of 1.2 mm × 0.5 mm × 0.5 mm (lateral × vertical × depth). The spatial resolution of the images has been reported to be 1.3 µm (isotropic, same in axial and lateral directions), allowing for cellular resolution imaging.^19^

The laser source used was a broadband supercontinuum laser emitting 3-nanosecond pulses at a repetition rate of 78 megahertz (MHz). The emitted spectrum at the output of the imaging probe had an approximately Gaussian shape, with a full width at half maximum (FWHM) of 230 nm and a central wavelength of λ = 820 nm, enabling high-resolution imaging across the full thickness of the cornea. The average power measured at the output of the DeepLive device, using a powermeter (PM100D with S130C sensor; Thorlabs, Newton, NJ), was 7.4 mW. Considering the average power and the repetition rate, the energy per pulse was therefore 9.5 × 10^−11^ J.

The DeepLive device is a CE-marked class IIa medical device and has a US Food and Drug Administration (FDA) 510(k) pre-market notification. In both cases, its intended use is for skin imaging. The system has been marked as a class I laser device according to the IEC 60825-1 standard (safety of laser products),^36^ taking into account that the function of the system does not involve intentional viewing over a long period.

The DeepLive device also includes white LED illumination at the distal end of the imaging probe, to provide illumination for a secondary imaging path allowing to obtain color surface images over a field of view of 2.6 mm, simultaneously to LC-OCT imaging. In the context of skin imaging, those images are also used to co-localize the LC-OCT images within larger field of view images obtained with another imaging device (dermatoscope). The accessible radiation of the LEDs is in the “exempt risk” group according to the IEC 62471 standard which applies to both skin and eye exposure.^36^ Regarding eye exposure, the exempt risk classification was determined by considering the blue light retinal hazard (main hazard considering the spectrum of the LED illumination, provided in Supplementary Fig. S1 for reference). A blue light weighted radiance of 7 W/m²/sr^−1^ was measured, below the limit of 100 W/m²/sr^−1^ for the “exempt” risk group (cf table 6.1 of the standard).

### Animals

Five 14-week-old female New Zealand White (NZW) rabbits (Charles River Laboratories, L'Arbresle, France) were used. Female rabbits were chosen to ensure animal welfare and experimental integrity, as male rabbits are highly territorial and may show aggressive behavior when housed together, leading to injuries and stress. Housing male with female rabbits would also risk uncontrolled breeding, introducing variability. All procedures involving animals were conducted in accordance with the ARVO Statement for the Use of Animals in Ophthalmic and Vision Research. The experimental protocol was approved by the Ethics Committee of the Animal Facility (CEEAL-UJM N°98) at the University of Saint-Étienne and the French Ministry of Higher Education and Research (protocol no. 2024052416544471_v3).

### LC-OCT Imaging

The rabbits were anesthetized via intramuscular injection of ketamine (Clorketam 100; Vetoquinol, Lur, France) at a dose of 35 mg/kg and xylazine (Rompun 2%; Elanco, Sèvres, France) at 5 mg/kg. Local anesthesia was administered by applying 1% tetracaine eye drops (Novartis Pharma, Rueil-Malmaison, France). Mydriasis was induced by instilling a combination of tropicamide 2 mg/0.4 mL (Mydriaticum; Théa Pharma, Clermont-Ferrand, France) and phenylephrine hydrochloride 10% (Néosynéphrine; Europhta, Monaco) eye drops, applied 15 minutes before and during the administration of systemic anesthesia.

**Figure 2.**
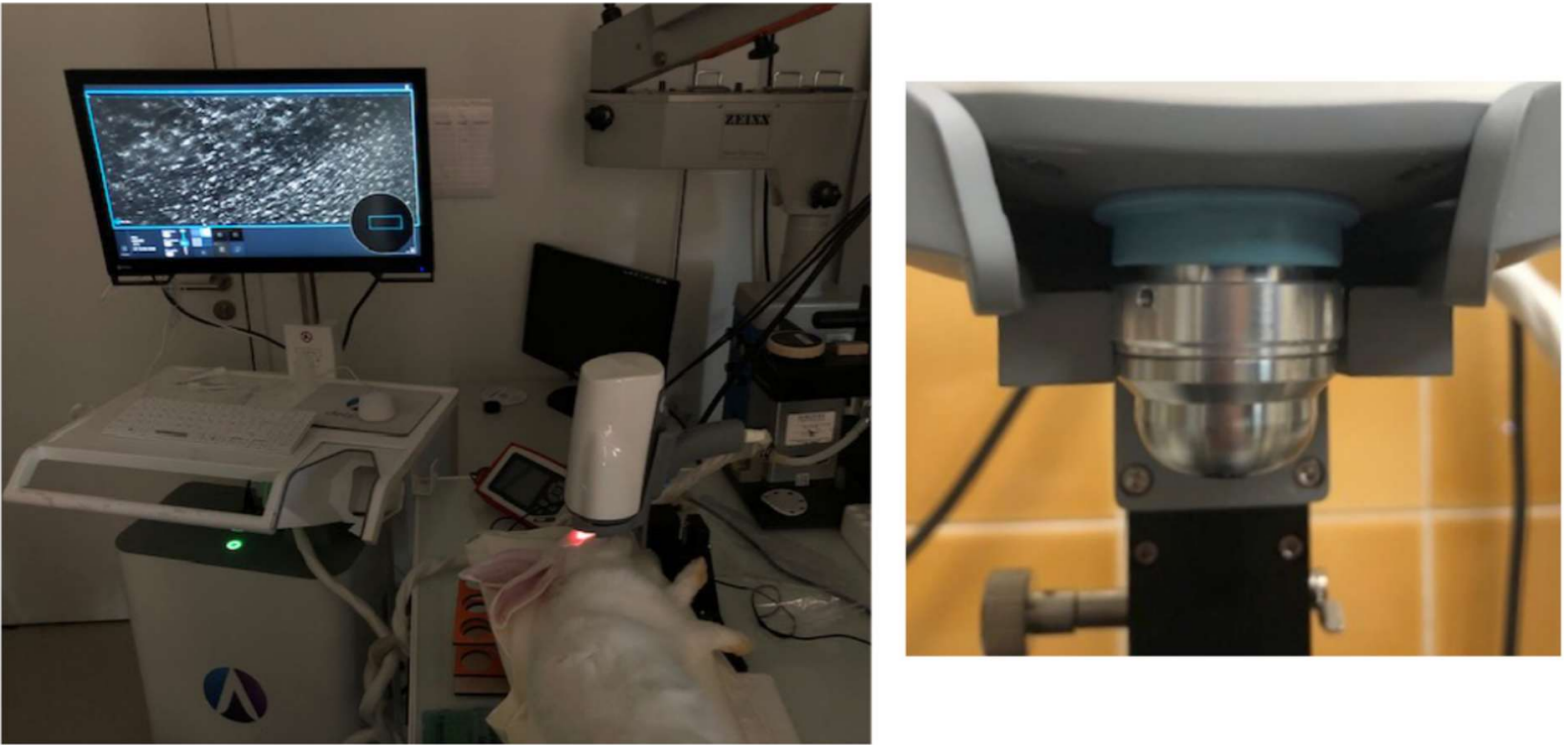
Experimental setup for in vivo LC-OCT imaging of the rabbit cornea using the DeepLive system. *Left*: Overview of the setup showing the DeepLive device with the probe positioned above the rabbit eye in a custom holder to ensure stable positioning during imaging. *Right*: Close-up view of the probe tip, which interfaces with the corneal surface for high-resolution imaging.

The cornea of one eye from each rabbit was imaged using the DeepLive device. The imaging probe was positioned in a custom holder with adjustable height in order to easily position the probe on the rabbit’s eye. During in vivo imaging in rabbits, the glass window at the tip of the DeepLive probe was placed in contact with the cornea with a layer of sterile artificial tear gel (Gel-larmes; Théa Pharma, Clermont-Ferrand, France) serving as the optical coupling interface (Fig. 2). No glass window was applied to the contralateral reference eye. The apex of the cornea was aligned with the center of the imaging lens to ensure full-thickness corneal imaging. Imaging sessions lasted 5 to 10 minutes per rabbit, which can be regarded as a worst case considering the usual durations of clinical examinations. The laser power was also increased from 7.4 mW to 13.2 mW, to represent a worst case in terms of laser power, taking into account the power fluctuations that can happen between DeepLive devices. In that case, the energy per pulse was 1.7 × 10^−10^ J. Figure 1B shows the spectral power density (SPD) measured at the output of the system i7 in those conditions. The measurement was performed using an integrating sphere connected to two calibrated spectrometers (FLAME-T and FLAME-NIR+; Ocean Optics, Orlando, FL). Three imaging modalities were used: (1) real-time 2D horizontal imaging to capture superficial views of corneal layers, (2) real-time 2D vertical imaging to visualize depth-resolved structures, and (3) 3D imaging to generate navigable volumes of the cornea across x, y, and z axes.

### In Silico Safety Evaluation

**Figure 3.**
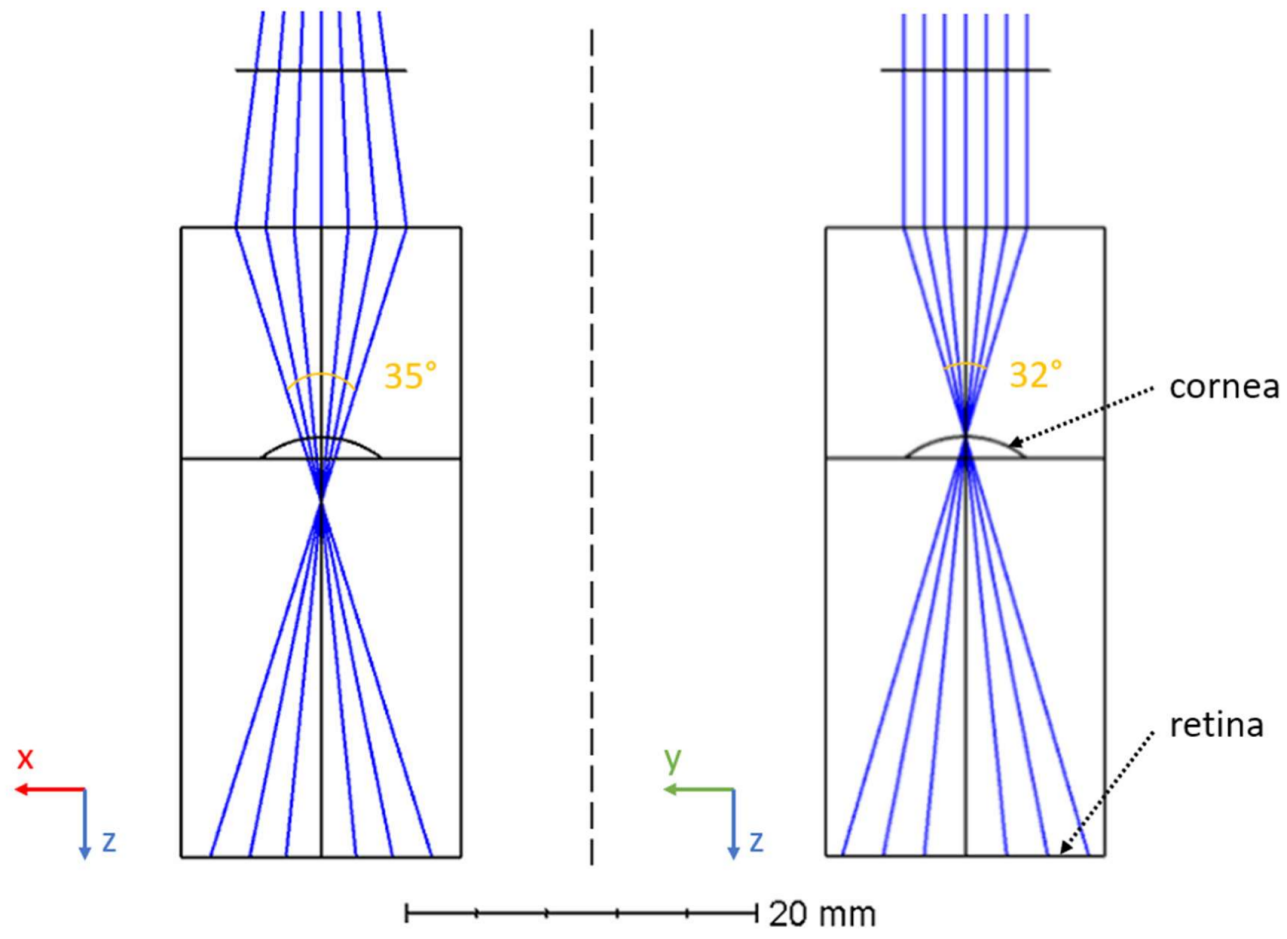
Schematic representation of the laser beam geometry at the output of the DeepLive device. The diagram illustrated the angular subtense of 35 degrees in the plane of the illumination line (x × z), and 32 degrees in the plane perpendicular to the illumination line (y × z), highlighting the divergent beam distribution used for corneal imaging in NZW rabbits.

Following the IEC 60825-1 standard, we evaluated the safety of the DeepLive device when used for ophthalmic imaging by comparing the output energy and power of the device to the maximum permissible exposures (MPEs) set by the standard, based on the wavelength range, emission duration, and beam geometry of the laser illumination. Considering the pulsed nature of the supercontinuum laser, and as required by the standard, both the energy per pulse and the average power were considered for comparison with the MPE. The geometry of the beam at the output of the DeepLive device has been evaluated from modeling the system using OpticStudio (Zemax, Kirkland, WA). Figure 3 showed a schematic of the illumination geometry. The angular subtense of the beam as defined by the IEC 60825-1 standard was 35 degrees in the plane of the illumination line (x × z), and 32 degrees plane perpendicular to the illumination line (y × z). This difference was due to the astigmatism introduced by the cylindrical lens. Considering this divergent geometry, table A.2 (MPE expressed in irradiance or radiant exposure) or A.4 (MPE expressed in power or energy) from the IEC 60825-1 standard must be taken into account for MPE evaluation, adapted for non-collimated lasers.

As the laser was a pulsed laser, 3 MPEs must be taken into account: the MPE of a single pulse, the MPE of a single pulse multiplied by a correction factor C_5_ if the pulses are shorter than 0.25 seconds, which was the case here, and the average MPE over the maximum exposure duration.

### In Vivo Safety Evaluation

In vivo safety evaluations were conducted at baseline (day 0 [D0]), immediately postoperatively, and on D7 and D14 (Supplementary Table S1). After general anesthesia, local anesthesia by 1% tetracaine eye drops and mydriasis as before LC-OCT observations, the following assessments were performed: (1) slit-lamp photography of the cornea and lens (SL-17, Kowa, Japan, equipped with a digital camera DG-GX880K, Panasonic, Japan); (2) retinal imaging obtained using a veterinary fundus camera (Epicam V, Epipole, Dunfermline, United Kingdom); and (3) intraocular pressure (IOP) measurement with a rebound tonometer (Tonovet; Icare, Vantaa, Finland).

Corneal transparency was quantified using a 5-stage scoring system, whereas neovascularization and inflammation were assessed with 4-stage scoring systems. Epithelial integrity was graded according to the Oxford score, a clinical standard for evaluating the severity and monitoring of dry eye disease.^37^ See Supplementary Table S1 for a full summary of the experimental conditions. The lens opacity was graded with the Lens Opacities Classification system III (LOCS III).^38^

On D7, the anesthesia protocol was modified to reflect the reduced handling time, which consisted solely of an imaging session. Ketamine was administered at a dose of 15 mg/kg, and medetomidine (NarcoStart; Ceva, Libourne, France) at 0.25 mg/kg, to ensure adequate but minimal sedation.

An injection of atipamezole hydrochloride (Atipam; Dechra, Montigny-le-Bretonneux, France) at a dose of 1 mg/kg, an alpha-2 adrenergic receptor antagonist, was administered immediately after the completion of clinical observations on D0 and D7 to facilitate and ensure the safe recovery of the animals.

### Postmortem Examinations

On D14, animals were euthanized by an overdose of pentobarbital sodium (Euthasol Vet.; Decrha, Montigny-le-Bretonneux, France) at 140 mg/kg by intravenous injection following general anesthesia induced by subcutaneous injection (<2 mL) of ketamine (35 mg/kg) and xylazine (5 mg/kg).

The corneas and retinas aligned with the optical axis from one control eye and 5 treated eyes were fixed in 4% paraformaldehyde for cross section histology. These samples were dehydrated through ascending concentrations of ethanol and embedded in paraffin. Cross sections 7-µm thick were cut, rehydrated, and stained with hematoxylin, eosin, and saffron (HES). Bright-field tagged image format file (TIFF) images of the cross sections were acquired using an IX81 microscope (Olympus, Tokyo, Japan). The histological sections were reviewed by an experienced ocular pathologist (author J.M.D.) who was masked to the treatment allocation; the slides were coded so that the pathologist could not identify whether they came from OCT-imaged eyes or control eyes.

### Statistical Analysis

IOP measurements were analyzed using a 2-way analysis of variance (ANOVA) to evaluate the effects of time and treatments. Specific differences between time points were assessed using Tukey’s post hoc tests. All statistical analyses were performed using GraphPad Prism software, version 10.

## Results

### Image Quality

**Figure 4.**
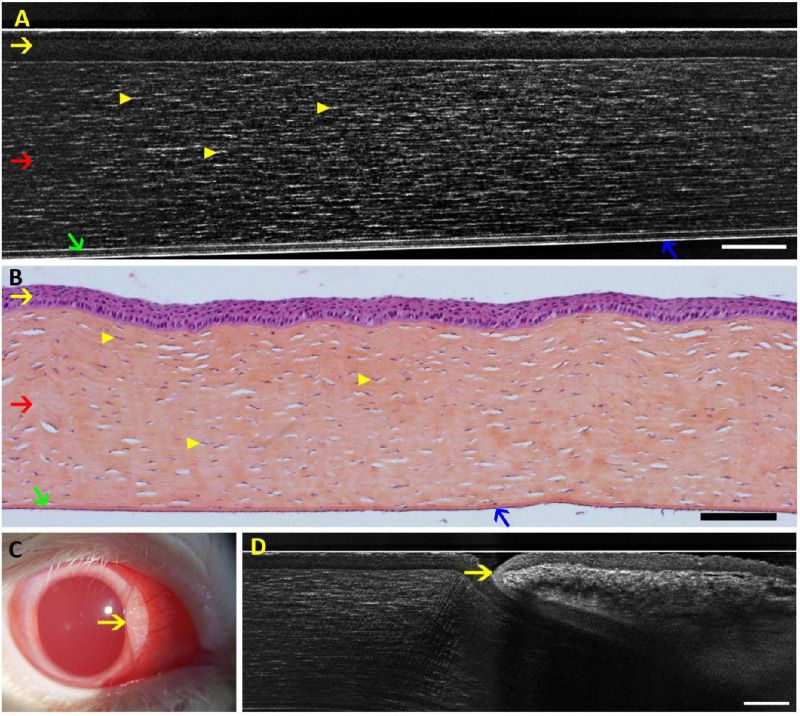
LC-OCT imaging of the rabbit cornea and cornea–nictitating membrane interface, with histological comparison. (**A**) LC-OCT cross-sectional image showing near-histological resolution. The *top white line* was the interface between the glass slide and the cornea. *Scale bar* = 100 µm. (**B**) Corresponding histological section observed with transmission light microscopy after hematoxylin, eosin, saffron (HES) staining. *Scale bar* = 150 µm. *Yellow arrow*: Corneal epithelium; *red arrow*: corneal stroma; *green arrow*: Descemet's membrane; and *blue arrow*: corneal endothelium. *Yellow arrowheads*: Keratocytes. (**C**) Slit-lamp en face view of the rabbit eye showing the relative position of the nictitating membrane. (**D**) LC-OCT cross-sectional image of the interface between the cornea (*left side* of the image) and the nictitating membrane (*right side*). *Scale bar* = 100 µm. *Yellow arrow*: nictitating membrane edge.

LC-OCT provided high-resolution images of the living rabbit cornea. In vertical cross-sectional views, similar to histological sections, all normal corneal structures could be identified. Although histological images provide superior cellular resolution within the epithelial layer, LC-OCT enables clear delineation of the epithelium without sectioning artifacts. In LC-OCT images, individual epithelial cells are only partially distinguishable, as visualization of superficial cells is limited by the highly reflective contact interface and basal cells remain difficult to resolve (Fig. 4). As expected in rabbits, no Bowman's membrane was observed.

**Figure 5.**
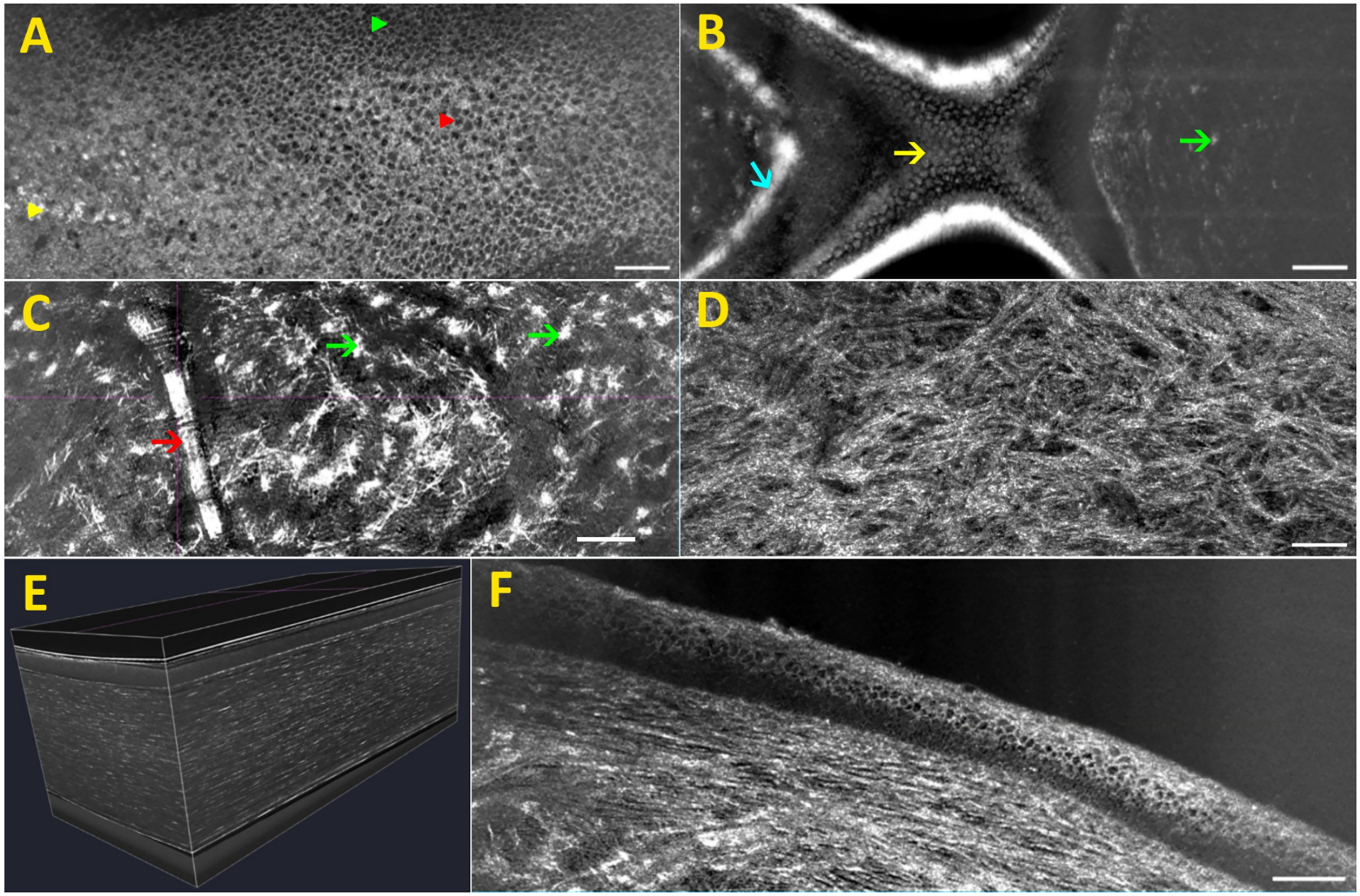
Multimodal LC-OCT imaging of the rabbit cornea and conjunctiva. (**A**) Horizontal view of the corneal epithelium. In this slightly oblique section, the small basal cells (*green arrowhead*) and larger intermediate cells (*red arrowhead*) were individualized. However, in the most superficial layer, only hyper-reflective superficial debris, probably corresponding to desquamating cells, were visible (*yellow arrowhead*). (**B**) Horizontal view of the corneal endothelium. (**C**) Horizontal view of the corneal anterior stroma. (**D**) Horizontal view of the conjunctiva. *Red arrow*: stromal nerve fiber; and *green arrows*: keratocyte cell bodies. *Blue arrow*: Descemet's membrane; *yellow arrow*: endothelial cells; and *green arrow*: keratocyte cell body. (**E**) Three-dimensional (3D) reconstruction of the rabbit cornea, enabling volumetric tissue exploration (see Supplementary Video S1). (**F**) Cross-sectional view of the conjunctiva, showing the distinct layers of the conjunctival epithelium. *Scale bar* = 100 µm for all images.

In horizontal views, the basal and intermediate epithelial cells were individualized; however, the superficial cells were not clearly visible (Fig. 5A). Within the corneal stroma, imaging enabled visualization of a nerve fiber, as well as numerous keratocytes appearing as hyper-reflective cell bodies (Fig. 5C). Cytoplasmic extensions (dendrites) were also visible, confirming the typical morphology of these stromal cells. At the posterior interface, Descemet's membrane appeared as a hyper-reflective boundary separating the stroma from the endothelial cell layer. The endothelial cells were clearly distinguishable from one another, allowing for detailed cellular analysis of the endothelium (Fig. 5B).

Additionally, LC-OCT imaging provided cross-sectional visualization of the conjunctiva (Fig. 5F), and enabled 3D reconstruction (cube view) of the cornea (Fig. 5E).

The integration of confocal and OCT modalities in the LC-OCT system allowed for high-resolution 3D imaging (1.2 mm × 0.5 mm × 0.5 mm), enabling dynamic tissue exploration in depth using the dedicated software.

### Safety

#### In Silico

Considering the polychromatic nature of the supercontinuum laser, and the additive nature of the wavelengths in the laser spectrum for eye hazard, in order to accurately take into account the MPE provided by the standard IEC 60825-1, it is necessary to divide the spectrum in a number of ranges of wavelength, and for each range, compute the ratio of the power or energy in that range to the MPE for this range. The sum of the ratios for all the ranges must not exceed unity to consider that the overall MPE is not exceeded (cf. section 4.3.b of the standard). In the following, the spectrum was divided in 20 ranges of 30 nm.

Considering the pulse duration of 3 ns and the illumination wavelength range, the MPE for a single pulse expressed in energy, according to table A.4 of the standard, was the following:

$$\begin{array}{c} \left\{\begin{array}{c} {\mathrm{MPE}}_{single\;pulse}=7.7\;\times \;10^{-8}C_{6}\;J\;\mathrm{for}\;\lambda \;=\;600\;\mathrm{to}\;700\;\mathrm{nm}\\ {\mathrm{MPE}}_{single\;pulse}=7.7\;\times \;10^{-8}C_{4}C_{6}\;J\;\mathrm{for}\;\lambda \;=\;700\;\mathrm{to}\;1050\;\mathrm{nm} \end{array}\right. \end{array}$$

As the angular subtense of the beam is larger to 100 mrad,

$$\begin{array}{c} C_{6}=\frac{\alpha_{max}}{\alpha_{min}}=3.33 \end{array}$$

as α_min_ = 1.5 mrad and α_max_ = 5 mrad, as the pulses duration is below 625 µs.

Furthermore,

$$\begin{array}{c} \left\{\begin{array}{c} C_{4}=1\;\mathrm{for}\;\lambda \;=\;600\;\mathrm{to}\;700\;\mathrm{nm}\\ C_{4}=10^{0.002\left(\lambda -700\right)}\mathrm{for}\;\lambda \;=\;700\;\mathrm{to}\;1050\;\mathrm{nm} \end{array}\right. \end{array}$$

**Figure 6.**
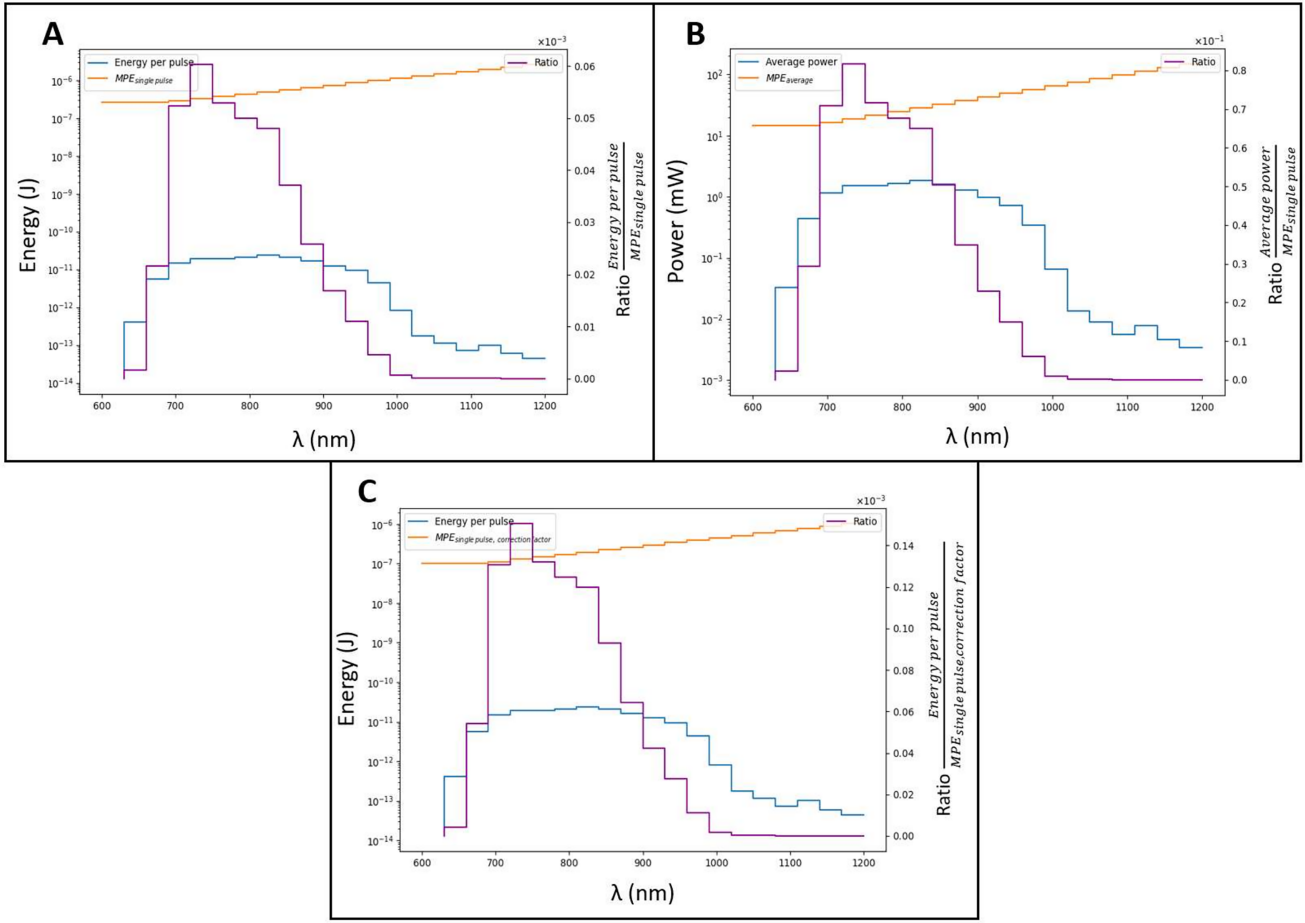
Maximum permissible exposure (MPE) analysis of the DeepLive device. (**A**) For a single pulse. (**B**) For a single pulse, with correction factor. (**C**) For the average power over 10 minutes.

The MPE_single pulse_, as a function of the wavelength, along with the energy per pulse computed for each 30-nm range from the SPD of the laser is shown in Figure 6. On this figure, the ratio between the energy per pulse and the MPE_single pulse_ is also shown for each range.

The sum of the ratios is 0.00038 < 1.

In our case, the maximum anticipated exposure duration is larger than 0.25 seconds, and the number of pulses of this duration is much above 600, therefore the correction factor C_5_ is 0.4.

Figure 6 shows the MPE_single pulse_ corrected by this factor, along with the energy per pulse and the ratio between the energy per pulse and the MPE_single pulse_ corrected by this factor for each 30-nm range.

The sum of the ratios is 0.00096 < 1, therefore, the MPE for a single pulse multiplied by a correction factor C_5_ is not exceeded.

The average MPE over the maximum exposure duration, considered to be t approximately 10 minutes, representative of the longest examination that could happen in practice, is expressed in power in table A.4 of the standard, considering that t > 100 seconds:

$$\begin{array}{c} \left\{\begin{array}{c} {\mathrm{MPE}}_{average}=\;7\;\times \;10^{-4}C_{6}T_{2}{\;}^{-0.25}\;W\;\mathrm{for}\;\lambda \;=\;600\;\mathrm{to}\;700\;\mathrm{nm}\\ {\mathrm{MPE}}_{average}=\;7\;\times \;10^{-4}C_{4}C_{6}T_{2}{\;}^{-0.25}\;W\;\mathrm{for}\;\lambda \;=\;700\;\mathrm{to}\;1050\;\mathrm{nm} \end{array}\right. \end{array}$$

with *T*_2_ = 100 seconds as the angular subtense of the beam is larger to 100 mrad. Let us note that the value of α_max_ is different from the previous calculation as t > 625 µs. Here, as *t* > 0.25 s, α_max_ = 100 mrad, therefore *C*_6_ = 66.67.

The MPE_average_ as a function of the wavelength, along with the power computed for each 30-nm range from the SPD of the laser, is shown in Figure 6. On this figure, the ratio between the power and the MPE_average_ was also shown for each range.

As the sum of the ratios was 0.52 (<1), we concluded that the average MPE over the maximum exposure duration was not exceeded.

In consequence, none of the 3 MPEs from the IEC 60825-1 standard were exceeded considering the characteristics of the DeepLive device illumination on the sample. The system could therefore be regarded as safe for ocular imaging according to the IEC 60825-1 standard.

#### In Vivo

**Figure 7.**
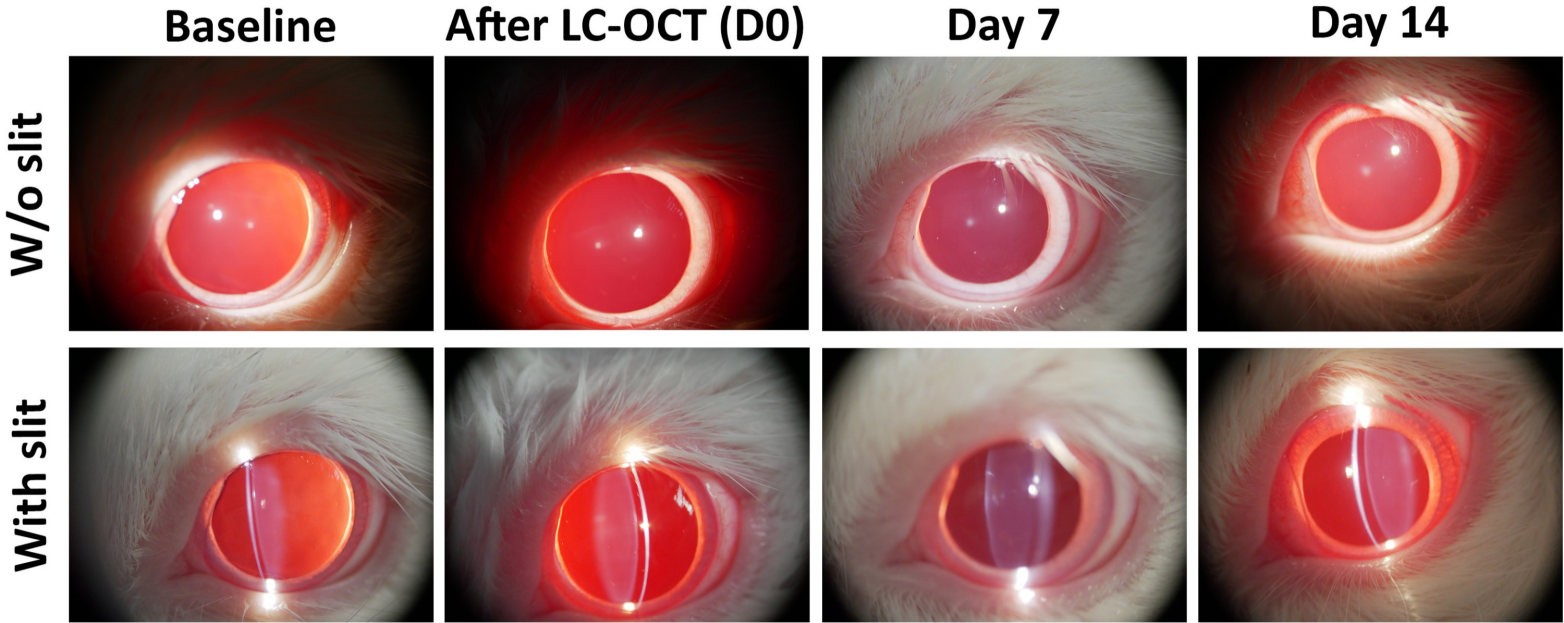
Slit-lamp observations of exposed rabbit eyes over time. Clinical slit-lamp photographs of an exposed rabbit eye before (baseline), immediately after, 7 days after, and 14 days after LC-OCT exposure. Images demonstrate sustained corneal clarity, lens transparency (LOCS III grade = 0), and intact epithelium throughout the 14-day follow-up period.

Regarding the cornea, no signs of opacification, neovascularization, or inflammation were detected in the 5 eyes exposed to the device at all follow-up points: before observation with the DeepLive device, immediately after the intervention, as well as on D7 and D14 (Fig. 7). According to our scoring table (see Supplementary Table S1), all scores remained at zero for all five animals throughout the entire duration of our study. Additionally, the integrity of the corneal epithelium was confirmed by the absence of fluorescein staining and an Oxford score of 0 on D14 (see Fig. 7). The transparency of the crystalline lenses in the experimental group was similar to that of the controls, with no notable differences. The LOCS grade remained at zero for the nuclear, cortical, and posterior subcapsular opalescence in all the eyes (see Fig. 7).

Concerning the IOP, there was no difference between exposed and control eyes (*P* = 0.6713). A significant difference between D7 and D14 for both groups (*P* = 0.0319) was observed (data not shown).

**Figure 8.**
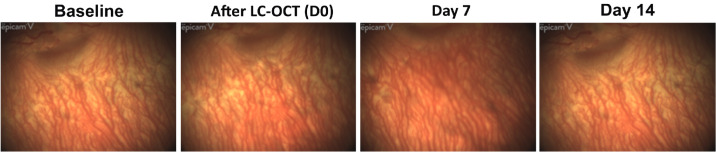
Fundus photography of exposed rabbit retina over time. Representative fundus photographs of an exposed rabbit eye's central retina along the optical axis at baseline, immediately after, 7 days after, and 14 days after LC-OCT exposure. Images show no signs of vitritis, retinitis, or optic neuropathy, indicating preserved retinal integrity.

Finally, macroscopic examination of the retina along the optical axis revealed no alterations in the exposed eyes, before or after observation with LC-OCT (Fig. 8).

**Figure 9.**
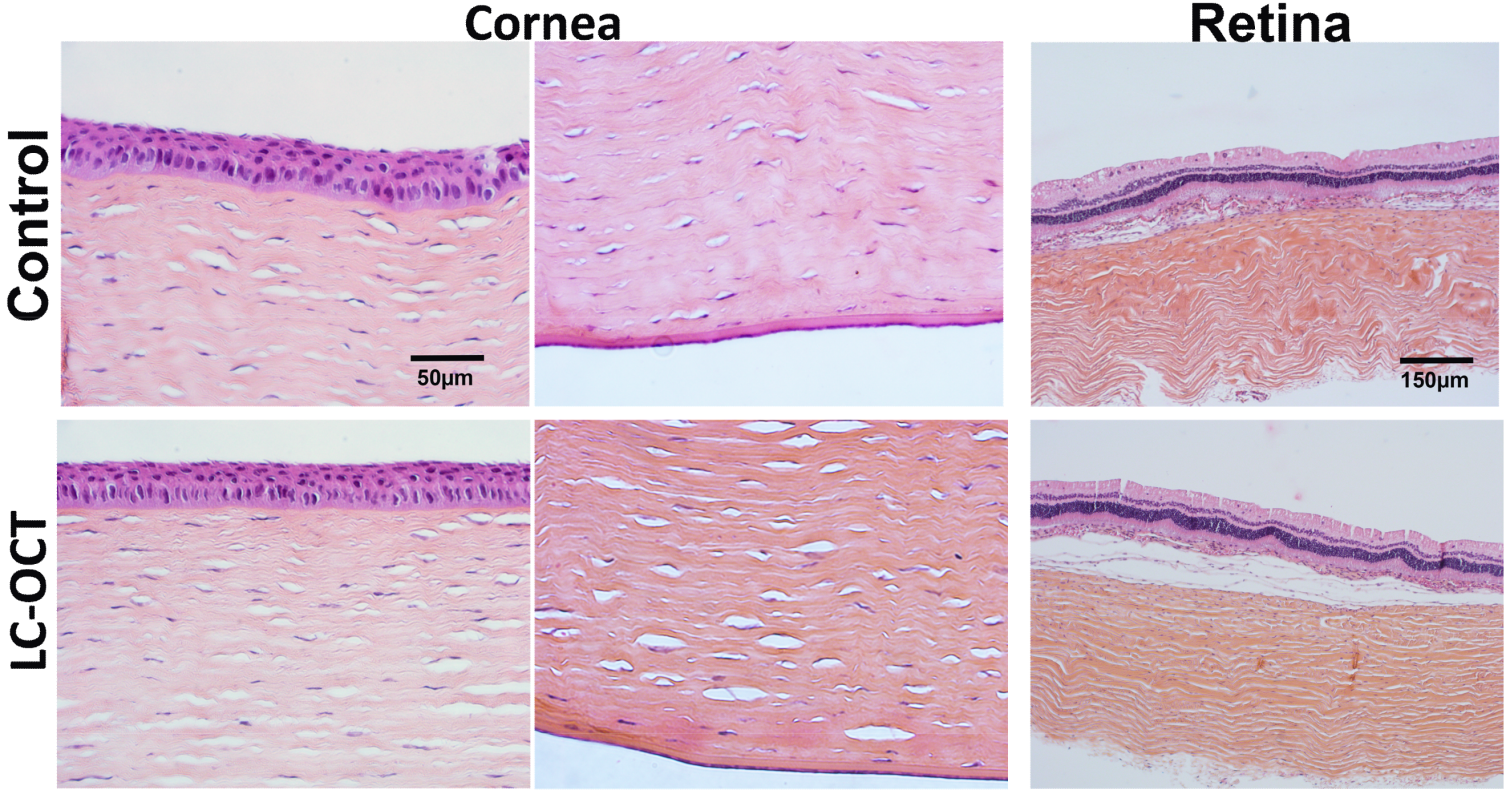
Histological sections of cornea and retina post-LC-OCT exposure. Hematoxylin, eosin, and saffron (HES)-stained cross-sections of LCO-OCT observed and control rabbit corneas (epithelium and endothelium) and retinas 14 days after LC-OCT exposure. Corneal sections reveal normal epithelial, stromal, and endothelial layers without edema or inflammation. Retinal sections display intact cellular layers with no gliosis, inflammation, or photoreceptor degeneration.

Histological sections confirmed the integrity of the ocular structures. The exposed and control corneas showed normally organized epithelial, stromal, and endothelial layers, with no signs of edema or inflammation (Fig. 9). Regarding the retina, all cellular layers were preserved in both treated and control eyes. No signs of disorganization, necrosis, inflammation, or degeneration of photoreceptors were observed (see Fig. 9). Two retinal detachments detected were identified as postmortem artifacts resulting from dissection.

## Discussion

This study reports in vivo ophthalmic using a commercially available, CE-marked LC-OCT system (DeepLive) to image the anterior segment of NZW rabbit eyes. Our findings confirm the feasibility and safety of this technology over a 14-day observation period, fulfilling the study's aim to evaluate its potential in ophthalmology. The absence of clinical or histological evidence of corneal, lens, or retinal damage underscores its safety profile, even under a worst-case scenario of 5 to 10 minutes of continuous exposure at 13.2 mW along the optical axis—far exceeding typical clinical imaging durations of 30 seconds to 1 minute. This extended exposure simulates potential misuse, providing a robust safety margin for future human applications.

Importantly, whereas previous studies have demonstrated the technical capabilities of LC-OCT for high-resolution imaging of biological tissues, these systems were primarily research prototypes or custom-built devices developed in laboratory settings. For example, early work by Lawman et al. demonstrated high-resolution corneal imaging using line-field spectral-domain OCT in an experimental configuration, highlighting the potential of the technology for ophthalmic applications.^34^ More recently, Neuhaus et al. reported real-time cellular-resolution imaging using line-field OCT systems designed for research purposes.^35^ In contrast, the present study evaluates, for the first time, the safety and feasibility of a commercially available, CE-marked LC-OCT system in an ophthalmic context. This distinction is important from a translational and regulatory perspective, as the use of a clinically approved device represents a critical step toward routine clinical implementation.

The NZW rabbit model was chosen for its established relevance in ophthalmic safety studies, owing to its ocular anatomy similar to humans and heightened sensitivity to phototoxicity.^39^^–^^41^ This selection enhances the translational validity of our results. The LC-OCT system illuminated a 1.2 mm × 1.3 µm line on the cornea, whereas defocused light was distributed over an approximately 9 × 18 mm area on the retina, thereby minimizing localized energy deposition. Supplementary Figure S2 shows the intensity distribution of the laser in the cornea and retina planes, simulated using OpticStudio and the Emsley reduced eye model to simulate the eye.^42^ Compliance with the IEC 60825-1 standard, with laser exposure below maximum permissible limits for single pulses, corrected pulse trains, and average power over 10 minutes, further corroborates the absence of phototoxicity, reducing risks of cataracts or retinal damage in brief, discontinuous clinical use.

A standout feature of LC-OCT is its ability to generate high-resolution, real-time 3D images, as evidenced by the volumetric “cube” of the cornea, layer-specific reconstructions (epithelium, stroma, and endothelium) and possibility to navigate across lateral, vertical, and depth axes, allowing detailed visualization of the anterior segment within a volume of 1.2 × 0.5 × 0.5 mm^3^.

AS-OCT, although effective for cross-sectional imaging, lacks cellular resolution and real-time 3D reconstruction,^1^ whereas IVCM provides cellular detail but is constrained by shallow depth, small field of view, and inability to produce 3D reconstructions,^7^ LC-OCT's integration of cellular resolution (1.3 µm) with depth-resolved 3D imaging, already transformative in dermatology,^19^ positions it as a potential successor to IVCM in ophthalmology and a complement to AS-OCT. The observed 3D views of corneal layers, alongside 2D cross-sections revealing epithelial cells, stromal collagen, and endothelial outlines, highlight its versatility for detailed structural analysis.

Potential clinical applications are broad. The high-resolution 2D and 3D images of the cornea, with a depth of 0.5 mm covering its full thickness, suggest utility in diagnosing pathologies like fungal or acanthamoeba keratitis, where cellular detail is critical.^9^^–^^11^ The ability of LC-OCT to visualize the conjunctival edge and the cornea–nictitating membrane junction highlights its potential for the diagnosis and monitoring of conjunctival and eyelid lesions, such as nevi, primary acquired melanosis (PAM), melanoma, and squamous cell carcinoma. This application could build on LC-OCT's proven success in dermatology for skin lesion assessment^20^^–^^23^ and may offer performance comparable to IVCM for these indications, in addition to its relevance for infectious and inflammatory diseases. This is particularly relevant at a time when no commercially available microscopic imaging device currently covers the anterior ocular segment. Furthermore, the safety demonstrated for anterior segment imaging also validates the expansion of the use of LC-OCT for skin lesions of the eyelid, including eyelid skin cancer, accounting for 5% to 10% of all skin cancers.^43^ Additionally, the real-time imaging capability of LC-OCT could enhance surgical guidance and longitudinal monitoring of anterior segment diseases, addressing diagnostic gaps left by AS-OCT and IVCM.

Despite generally very high image quality, this first application of a CE-marked LC-OCT for dermatology on the cornea highlights several areas for improvement, mainly in terms of resolution and contrast, as well as motion artifacts or those related to contact with the front glass slide. Respiratory and cardiac movements in anesthetized rabbits introduced motion artifacts. Furthermore, in our experimental setting, imaging sessions involved prolonged acquisitions aligned with the optical axis. In contrast, clinical LC-OCT examinations are usually brief and performed slightly off-axis, allowing patients to maintain fixation more easily and minimizing eye movements, which contributes to improved image clarity and fewer motion artifacts. The artificial tear gel interface, although effective, caused minor optical distortions due to viscosity and uneven application. Saline or other coupling agents could represent potential improvements.

Image quality could also be further improved by removing the front glass window, which would eliminate an additional optical interface and potentially reduce reflection-related artifacts.

Limitations warrant consideration. The 14-day observation period, whereas sufficient for acute safety, precludes assessment of long-term lens effects, although exposure parameters and prior studies suggest minimal risk at these wavelengths.^44^ The absence of electroretinography limits functional retinal safety confirmation, despite intact histology and low retinal energy deposition. IOP variations between D7 and D14, attributed to the ketamine-xylazine protocol's known IOP-lowering effect,^45^ were unrelated to LC-OCT exposure, reinforcing its benign profile. Finally, whereas the 3D cube and multi-axis views are a leap forward, their full diagnostic utility—for example, quantifying layer thickness or detecting subtle pathologies—requires further exploration in diseased models or humans.

## Conclusions

This study provides critical insights into the safety and feasibility of a commercially available, CE-marked LC-OCT system for ophthalmic imaging, supporting its future clinical translation. LC-OCT's ability to deliver real-time, high-resolution, 3D imaging of anterior segment structures surpasses conventional modalities, heralding its potential as a transformative tool for ophthalmic diagnostics. Furthermore, by compliance to IEC 60825-1 safety standards and by systematically evaluating ocular tissue integrity in NZW rabbits following LC-OCT exposure, we demonstrate that the DeepLive device induces no harmful effects, even under a worst-case scenario exceeding typical clinical conditions. The absence of damage to corneal, lens, or retinal structures supports its minimal risk profile for human ocular tissues. These findings establish a foundation for future human trials, advancing LC-OCT from a dermatological success to a promising innovation in ophthalmology.

## Supplementary Material

Supplement 1

Supplement 2

Supplement 3

Supplement 4
