# Supplementary figures and images for: Preclinical Safety and Feasibility Study of Line-Field Confocal Optical Coherence Tomography for Ophthalmology Applications
Source: Transl Vis Sci Technol. 2026 Jul 1;15(7):1. doi: 10.1167/tvst.15.7.1 (PMC13332527; doi:10.1167/tvst.15.7.1)

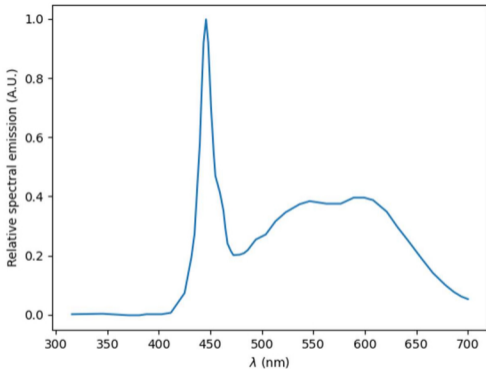

**Figure S1. Spectrum of the LED illumination at the distal end of the imaging probe.**

Supplement: Supplement 1 [file tvst-15-7-1_s001.pdf]
