# Supplementary material for: Preclinical Safety and Feasibility Study of Line-Field Confocal Optical Coherence Tomography for Ophthalmology Applications
Source: Transl Vis Sci Technol. 2026 Jul 1;15(7):1. doi: 10.1167/tvst.15.7.1 (PMC13332527; doi:10.1167/tvst.15.7.1)
Supplement: Supplement 2 [file tvst-15-7-1_s002.pdf]

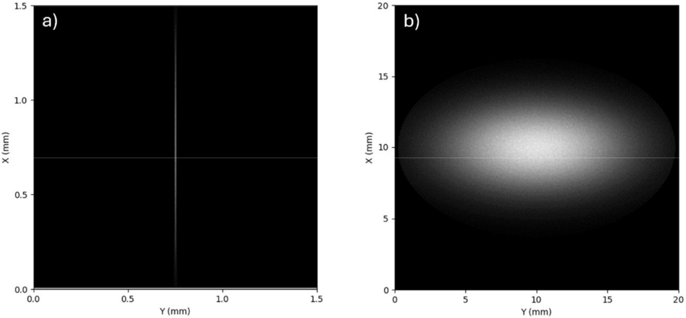

Figure S2. a) Intensity distribution of the laser in the plane of the cornea (line illumination). b) Intensity distribution of the laser in the plane of the retine (defocused line illumination).
