# Supplementary material for: Preclinical Safety and Feasibility Study of Line-Field Confocal Optical Coherence Tomography for Ophthalmology Applications
Source: Transl Vis Sci Technol. 2026 Jul 1;15(7):1. doi: 10.1167/tvst.15.7.1 (PMC13332527; doi:10.1167/tvst.15.7.1)
Supplement: Supplement 3 [file tvst-15-7-1_s003.pdf]

Table S-I. Scoring systems for corneal and ocular assessments. Criteria for quantifying corneal transparency, neovascularization, and inflammation levels in New Zealand White rabbits post-LC-OCT exposure, with scores ranging from 0 to 4 or 0 to 3.

**Transparency Level**	**Score**
Normal transparency, no opacity, all iris details are visible	0
Slight corneal haze. Iris details remain clearly visible.	1
Discernible opaque areas. Iris details become difficult to observe.	2
Severe corneal opacity. No iris details are visible; the pupil is barely discernible.	3
Complete corneal opacity. Iris and pupil are not visible.	4
**Neovascularization Level**	**Score**
No corneal neovascularization	0
Neovessels extending to the extreme periphery of the cornea (up to 1 mm from the limbus)	1
Neovessels extending into the peripheral cornea (up to 3 mm from the limbus)	2
Neovessels penetrating the central cornea (up to the center)	3
**Inflammation Level**	**Score**
No inflammation. Absence of Tyndall effect*, retrodescemetic precipitates (RDP), or iridolenticular synechiae	0
Moderate inflammation: Tyndall + and/or RDP +; no iridolenticular synechiae	1
Significant inflammation: Tyndall ++ and/or RDP ++; iridolenticular synechiae present	2
Severe inflammation: hypopyon or fibrin invading the anterior chamber	3

*Tyndall effect indicated anterior chamber flare.
